# Examining the relation between oral contraceptive use and attentional engagement in everyday life

**DOI:** 10.3389/fnhum.2023.1147515

**Published:** 2023-06-01

**Authors:** Alyssa C. Smith, Jeremy Marty-Dugas, Daniel Smilek

**Affiliations:** ^1^Department of Psychology, University of Waterloo, Waterloo, ON, Canada; ^2^Department of Psychology, Neuroscience and Behaviour, McMaster University, Hamilton, ON, Canada

**Keywords:** oral contraceptives (OCs), mind wandering, everyday attention, attention lapses, cognitive control

## Abstract

Oral contraceptives (OCs) used by women worldwide include artificial estradiol and progesterone, which can attach to receptors in the brain and potentially influence cognition. In the present studies, we examined the relation between OC use and self-reported everyday attention. We collected trait-level measures of mind wandering, attention-related errors, and attention lapses in undergraduate women using OCs (Study 1: OC group *N* = 471, Study 2: OC group *N* = 246) and naturally cycling women not using any form of hormonal contraceptives (Study 1: Non-OC group *N* = 1,330, Study 2: Non-OC group *N* = 929). In Study 1, we found that women using OCs reported significantly less spontaneous and deliberate mind wandering than naturally cycling women and no differences between groups on attention-related errors and attention lapses. In Study 2, our findings indicated no significant differences between groups on any of our attention measures. Regression analyses controlling for depression symptoms and semester of data collection found that OC use did predict unique additional variance on some attention measures, but these effects were small and unreliable across the two studies. Taken together, our data suggests there is little evidence that OC use is related to differences in attentional engagement in everyday life.

## Introduction

Worldwide, more than a hundred and fifty million women are currently taking oral contraceptives (OCs; United Nations, 2019). Most forms of OCs contain the synthetic hormones ethinyl estradiol and progestin, which mimic the naturally occurring female hormones estrogen and progesterone, respectively. In naturally cycling women, endogenous levels of estrogen and progesterone fluctuate over the course of the menstrual cycle, such that they are lower in the first half than in the second half of the cycle. However, in those using OCs, endogenous estrogen and progesterone are suppressed by the presence of exogenous estrogen and progesterone. Thus, those using OCs have much lower levels of endogenous estrogen and progesterone than those who are naturally cycling and maintain a consistent level of these *exogenous* hormones throughout most of the menstrual cycle by taking a pill each day. While the main goal of OC treatment is to influence the reproductive organs, receptors for estrogen and progesterone are distributed throughout the body, including in the brain (Brinton et al., 2008; Morissette et al., 2008). This suggests that OCs could influence many physiological systems, including various brain processes such as attentional engagement. In the present studies, we extend prior work by examining whether women using OCs and naturally cycling women differ in terms of their reports of their everyday attention abilities.

Previous research has suggested that use of OCs may be linked to increased inattention in everyday life (Raymond et al., 2019). Along these lines, Raymond et al. (2019) assessed levels of mind wandering in women using OCs (*N* = 28), naturally cycling women in the luteal phase (*N* = 14; naturally cycling women in the luteal phase were chosen because these women were most hormonally dissimilar to women using OCs. That is, women in the luteal phase tend to have higher levels of endogenous estrogen and progesterone, while women using OCs have low levels of endogenous estrogen and progesterone and high levels of exogenous estrogen and progesterone), and men (*N* = 29). Mind wandering was indexed using the short Imaginal Process Inventory (SIPI; Huba and Tanaka, 1983), which is a subjective report questionnaire that assesses three aspects of day dreaming/mind wandering, namely, (1) positive-constructive daydreaming, (2) guilt and fear-of-failure daydreaming and (3) poor attention control. They found no significant differences between women using OCs, naturally cycling women, and men on the guilt and fear-of-failure subscale or the positive-constructive daydreaming subscale of the SIPI. However, they found significant differences between groups on the attentional control subscale, such that women using OCs reported significantly poorer attention control than naturally cycling women and men. To rule out the possibility that these differences were due to differences in depression across groups—since inattention and depression are related (Smallwood et al., 2007; Carriere et al., 2008; Seli et al., 2019)—the authors showed that there were no significant differences among groups with regard to scores on the Beck Depression Inventory (BDI; Beck et al., 1988). It is worth noting, however, that scores on the BDI were associated with lower scores on the attentional control subscale of the SIPI. Unfortunately, the small sample size of the study precludes any strong conclusions about the relation between OC use and inattention.

Although our main focus here is the relation between OC use and attention abilities, given the well-established link between inattention and affective dysfunction (Carriere et al., 2008; Smallwood and O’Connor, 2011; Poerio et al., 2013; Seli et al., 2019), it is important to further consider the link between OC use and affect. On this issue, there has been much debate in the literature, with some studies reporting that OC use does not increase negative affect (Duke et al., 2007; Cheslack-Postava et al., 2015), others reporting that OC use is associated with increased positive mood (Toffol et al., 2012; Keyes et al., 2013; Hamstra et al., 2017), and still other studies reporting that OCs have a detrimental effect on affect (Skovlund et al., 2016). In addition, there is speculation that the link between OC use and depression symptoms might be mediated by various individual characteristics; for instance, individuals with a history of depressive symptoms may be particularly at risk of developing mental health problems as a result of OC use (Wiréhn et al., 2010; Lewis et al., 2019). It is also worth noting that studies on OC use may be confounded by a “survivor effect.” That is, individuals who experience adverse mood effects (or perhaps any adverse effect) as a result of using OCs tend to discontinue the medication and so are excluded from the OC use group, which artificially increases mean levels of mood for the remaining (i.e., “surviving”) OC users.

### The present studies

Here we present two studies that extend prior work on the relation between attentional engagement in everyday life and OC use. Study 1 had several primary aims. First, we aimed to examine the relation between OC use and attention by recruiting a larger sample of women than has been used in prior related studies. This is important given that prior studies examining this issue have relied on rather small samples [e.g., 28 women using OCs, 14 naturally cycling women in Raymond et al., 2019]. Second, while prior work has focused on daydreaming, we aimed to more specifically assess a wide range of everyday attention abilities by using well-validated self-report questionnaires that index spontaneous and deliberate mind wandering (Carriere et al., 2013), attention lapses (Carriere et al., 2008), and attention related errors (Cheyne et al., 2006; Carriere et al., 2008). Third, because of the established relation between OC use and mood, we also included a measure of depressive symptoms and considered this measure when assessing the relation between OC use and trait attention. In Study 2, we sought to replicate our findings from Study 1 using an independent sample of undergraduate women.

## Study 1

### Methods

This study was reviewed and received ethics clearance through a University of Waterloo Research Ethics Committee (ORE# 41701). The study was conducted in accordance with relevant guidelines and regulation. All participants provided informed consent. Data was collected online at the University of Waterloo at the beginning of three semesters during the 2020 calendar year: Winter 2020 (January-February), Spring 2020 (May-June), and Fall 2020 (September-October).

#### Prescreen data

The data collected for this study were a part of a larger test battery (Mass Testing). Mass Testing is administered online during the first 8 weeks of each semester using Qualtrics survey software. Students enrolled in undergraduate psychology courses at the University of Waterloo were eligible to complete this survey. This enabled us to survey a large number of females currently using or not using oral contraceptives. Participants also complete a separate prescreen survey (the Mass Testing and Pre-screen surveys are typically completed in close succession at the beginning of each semester) in which they were asked to provide their biological sex and to answer the following question about hormonal birth control use: “Are you currently using one of the following methods of birth control?” Participants responded by selecting from a list that included: oral contraception (i.e., birth control or “the pill”), birth control patch, vaginal ring, birth control injection, IUD, hormonal implant, none of the above (in Winter 2020 this option was “does not apply to me”), and prefer not to answer. Participants were also asked to specify whether they were currently being treated for depression or anxiety, whether they were currently using medications for psychosis, and the date of their last menstruation.

#### Data cleaning

In this section, we describe the basic data cleaning that was performed on all samples prior to computing descriptive and inferential statistics. For all semesters, the cleaning process was virtually identical (the associated R code can be found https://osf.io/dpxtn/). The Mass Testing survey is administered by the department (rather than by individual researchers), and as a result, sometimes there are changes/additions to the survey that are outside of researchers’ control. For example, in one sample, the response options to our question about the use of hormonal birth control were different than in the other samples (i.e., in Winter 2020 participants were asked to indicate their non-use of this medication with the option “does not apply to me,” while in subsequent samples this option was changed to “none of the above”; we treated these options as equivalent in the analyses). These changes account for most (if not all) of the differences in the cleaning code for the different samples.

After selecting the relevant variables from the Mass Testing survey (the Mass Testing survey Winter 2020 had two versions and as such, only half of the Mass Testing participants completed the version of the survey containing the scales of interest for the present study), we began by making standard exclusions for birth control research, such as excluding participants who indicated their biological sex was male and reported currently receiving treatment for depression, anxiety, or psychosis [similar to Raymond et al. (2019), Bradshaw et al. (2020)]. Further, given that in the present study we were interested in OC use, we also excluded participants who indicated they were using a different form of hormonal contraceptives (e.g., IUDs, injections, birth control patch, or vaginal ring). Participants who declined to indicate whether or not they were using a form of hormonal contraceptives were also excluded.

Next, we employed additional data cleaning procedures to ensure high quality data in participant responses. This is because the Mass Testing survey is a lengthy questionnaire, and many participants may not remain fully attentive over the course of the survey. In addition, we aimed to address the possibility that some participants may employ assistive technology, or “bots,” to help them receive their participation credit with less time and effort. After selecting the relevant items for each attention variable, we used timing data associated with each scale to help identify suspicious data. We examined three aspects of the data: (1) click data (how many clicks participants make on a given page of the survey), (2) the response timing data (the length of time participants spent on a given page of the survey), and (3) the number of items completed for each the scale.

First, we examined participants clicking behavior on each scale of interest. Each of our scales of interest was presented on a single page and Qualtrics logs the number of clicks each participant makes on each page of the survey, thus Qualtrics logged how many clicks participants made on each scale. We flagged responses as suspicious if participants made fewer clicks on a page than the number of scale items on the page. This method could catch three types of responses: (1) Participants who did not answer the scale at all (e.g., they answered none of the items on a scale containing four items, which resulted in zero clicks and four “NA” responses); (2) Participants who answered most, but not all, of the items in a scale (e.g., they answered three out of four items on the scale, resulting in three clicks and one “NA”); and (3) Participants who had too few clicks, but nonetheless had a score for every item (e.g., one click but four scores and 0 “NA” responses). We considered this third type of response to be suspicious—it is impossible to answer four questions with one click—and an indicator that the participant may have been assisted by a bot when filling out the scale. As such, because of the suspected bot use, we discarded the data from the participants with this third type of response (i.e., those with suspicious clicking behavior). In addition, we examined whether participants first and last click on the scale took place simultaneously. If a participant answered all the items simultaneously (i.e., the first and last click were simultaneous and the participant had no NAs for the scale), this may also indicate the use of a bot. As such, we removed participants who had simultaneous clicks.

Next, we examined participants’ timing data for each inattention scale. We decided that participants would need to have *at least* one second per item of a scale in order to read and answer the items appropriately. For each scale, we calculated the time taken to complete the scale by subtracting the time of their first click from the time of their last click on the page (i.e., the click to submit their responses for that scale). Participants whose time taken to complete the scale was equal to or less than the number of items in the were flagged as “too fast” (e.g., taking 6 s to answer a 12-item scale). Participants who were found to be “too fast” on two or more of the inattention scales (>50% of the scales) were removed from the analyses.

Finally, since scale scores were calculated by averaging items, we ensured participants’ scale scores were based on responses to the majority of scales items (i.e., participants did not leave most of the scale items blank). If, for example, a participant answered only two items of a 12-item scale, their scores of that scale would be less reliable and accurate than another individual who answered all 12 items. Since two of our scales of interest consisted of four items, we decided to exclude participants who had more than two “NA”s (non-responses) on any our inattention scales or our measure of depression.

After the data cleaning was completed for each semester, we created a list of those participants who were included. Following this, we cross-referenced the list of participants with those included from previous semesters to ensure that the participants included were independent from those previous semesters. This cross-reference procedure progressed sequentially through the semesters and those who had already participated were removed (i.e., Winter 2020 was cleaned first, then Spring 2020 was cross-referenced against Winter 2020, Fall 2020 was cross-referenced against Winter 2020 and Spring 2020). Partly as a function of this, our sample sizes decrease over time—as there were fewer new participants who had yet to be included in one of our prior semesters.

Participants who were removed during this cleaning process for one semester were still eligible to be included in subsequent semesters should they subsequently meet the inclusion criteria. For example, if a participant was removed for responding too quickly in Winter 2020, her data could still be included in Fall 2020 if her responses were slower. That is, responding to questions too quickly in an earlier semester did not preclude that participant from inclusion in a later semester.

#### Participants

Participants in our study were undergraduate students who indicated their sex as female. Mass Testing was completed in exchange for partial course credit. Exclusions from each semester were made using the criteria outlined in the Data Cleaning section above. The Winter 2020 cohort initially consisted of 1,075 females, of which 530 participants were excluded. Thus, 545 participants were retained in Winter 2020 with 167 individuals using OCs and 378 naturally cycling and not using any form of hormonal contraceptives. The Spring 2020 sample consisted of data from 861 females. Of these, 512 participants were excluded leaving 349 participants in the analysis (98 using OCs and 251 naturally cycling). The Fall 2020 cohort initially included 1,724 females, of which 817 were excluded leaving 907 participants (206 OC users and 701 naturally cycling women). Altogether, in Study 1 we included 1,801 participants in total, with 471 using OCs and 1,330 naturally cycling participants who were not using any form of hormonal contraceptives.

Participants in this final sample had an average age of 20.24 years. Twenty-nine participants did not provide a birth year from which we could calculate their approximate age^1^. While all participated indicated their sex as female, participants also reported their gender identities. Most reported being cis-gendered females (*N* = 1772). Other gender identities included non-binary (*N* = 15), two-spirited (*N* = 1), and male (*N* = 2)^2^. Eleven participants did not provide a gender identity.

#### Materials

##### Spontaneous (MWS) and deliberate (MWD) mind wandering scales

The MWS is a measure of spontaneous or unintentional mind wandering, whereas the MWD is a measure of deliberate or intentional mind wandering (Carriere et al., 2013). These self-report questionnaires consist of 4 statements each; representative examples include, “I allow my thoughts to wander on purpose” (MWD), and “I find my thoughts wandering spontaneously” (MWS). Each participant responds on a seven-point scale that ranges from 1 (*rarely*) to 7 (*a lot*). Higher scores indicate a greater tendency to engage in mind wandering (deliberate or spontaneous) in everyday life.

##### Mindful attention awareness scale-lapses only (MAASLO)

The MAASLO (Carriere et al., 2008) is a revised version of the MAAS (Brown and Ryan, 2003) which includes 12 items related to mindlessness or attention lapses in everyday life. Participants respond to items such as “I rush through activities without being to really attentive them” on a scale from 1 (*almost never*) to 6 (*almost always*). Higher frequencies of attention lapses are reflected as higher scores on the scale.

##### Attention-related cognitive errors scales (ARCES)

The ARCES measures the tendency to experience performance errors caused by attention lapses (Cheyne et al., 2006; Carriere et al., 2008). Participants rate items such as “I have to go back to check whether I have done something or not (e.g., turning out lights, locking doors)” on a scale from 1 (*never*) to 5 (*very often*). Higher scores indicate a greater frequency of cognitive errors.

##### Depression anxiety stress scale-21 (DASS-21)

The DASS is a 21-item measure assessing symptoms of depression, anxiety, and stress over the previous week (Antony et al., 1998). Participants rate items such as “I felt that I had nothing to look forward to” (depression), “I was worried about situations in which I might panic and make a fool of myself” (anxiety), and “I found it hard to wind down” (stress) on a scale from 0 (*did not apply to me at all*) to 3 (*applied to me very much or most of the time*). The scale contains seven items related to each of depression, anxiety, and stress and higher scores on the scale items indicate higher levels of these experiences.

## Results and discussion

All analyses were performed in R Core Team (2019). We used the psych, lme4, car, apaTables, and basic R packages to perform the Null Hypothesis Significance Tests. Anonymized data and analysis scripts will be available at https://osf.io/dpxtn/.

### Descriptive statistics

**FIGURE 1:**
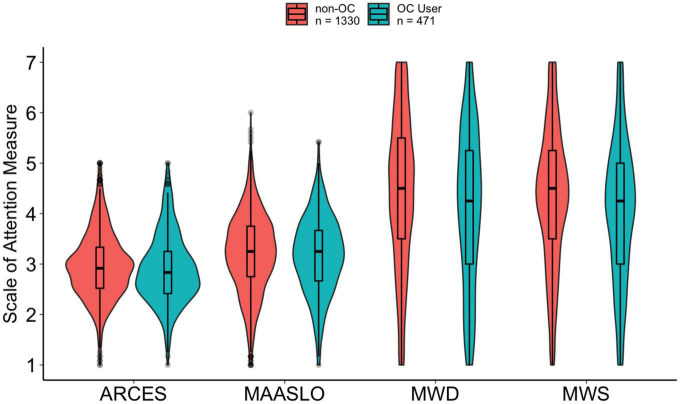
Violin plots with box and whisker plots (boxplots) for each of the attention measures [spontaneous mind wandering (MWS), deliberate mind wandering (MWD), mindful attention awareness scale (MAASLO), and attention-related cognitive errors scale (ARCES)] as a function of group in Study 1.

Descriptive statistics for both groups are provided in Table 1. All scales showed high reliabilities, with Cronbach alphas of 0.75 or greater. Cronbach alpha values and Pearson correlations between the measures within each group are provided in Supplementary Appendixes A1, 2. Boxplots depicting each of the attention measures as a function of OC use condition (averaged across semesters) are shown in Figure 1.

**TABLE 1:** Descriptive statistics of measures by semester and group (Study 1; *N* = 1,801).

Semester	Group	Measure	*N*	Mean	SD	Skew	Kurtosis
Winter 2020	Non-OC group	ARCES	378	2.95	0.63	0.14	0.39
		MAASLO	378	3.23	0.75	–0.14	0.06
		MWS	378	4.33	1.28	–0.09	–0.12
		MWD	378	4.33	1.41	–0.33	–0.36
		DASS-Dep	378	0.82	0.73	0.87	–0.12
		DASS-Anx	378	0.73	0.64	0.90	–0.02
		DASS-Stress	378	0.93	0.66	0.52	–0.46
	OC group	ARCES	167	2.88	0.62	0.64	0.71
		MAASLO	167	3.17	0.72	0.01	–0.10
		MWS	167	4.04	1.30	–0.12	–0.09
		MWD	167	4.04	1.48	–0.19	–0.73
		DASS-Dep	167	0.70	0.65	1.26	1.12
		DASS-Anx	167	0.63	0.58	1.12	1.11
		DASS-Stress	167	0.96	0.67	0.87	0.38
Spring 2020	Non-OC group	ARCES	251	2.84	0.63	0.11	0.39
		MAASLO	251	3.03	0.81	–0.08	–0.14
		MWS	251	4.00	1.41	–0.12	–0.59
		MWD	251	4.09	1.49	–0.06	–0.66
		DASS-Dep	251	0.78	0.65	0.91	0.03
		DASS-Anx	251	0.60	0.51	1.04	0.74
		DASS-Stress	251	0.85	0.58	0.67	0.15
	OC group	ARCES	98	2.77	0.65	0.46	0.43
		MAASLO	98	3.06	0.78	–0.04	0.32
		MWS	98	3.94	1.39	–0.06	–0.38
		MWD	98	3.96	1.54	–0.19	–0.59
		DASS-Dep	98	0.71	0.66	1.32	1.36
		DASS-Anx	98	0.58	0.50	0.89	0.47
		DASS-Stress	98	0.88	0.59	1.14	1.74
Fall 2020	Non-OC group	ARCES	701	3.03	0.66	0.42	0.26
		MAASLO	701	3.26	0.78	–0.10	0.23
		MWS	701	4.38	1.37	–0.29	–0.23
		MWD	701	4.62	1.54	–0.39	–0.48
		DASS-Dep	701	0.89	0.75	0.91	0.09
		DASS-Anx	701	0.74	0.64	1.06	0.76
		DASS-Stress	701	1.02	0.68	0.44	–0.41
	OC group	ARCES	206	2.96	0.73	0.34	0.07
		MAASLO	206	3.23	0.77	–0.30	0.14
		MWS	206	4.13	1.47	–0.17	–0.46
		MWD	206	4.31	1.59	–0.30	–0.81
		DASS-Dep	206	0.81	0.73	1.01	0.27
		DASS-Anx	206	0.75	0.66	0.95	0.27
		DASS-Stress	206	1.04	0.69	0.61	–0.18

MWS, spontaneous mind wandering; MWD, deliberate mind wandering; MAASLO, mindful attention awareness scale; ARCES, attention-related cognitive errors scale; DASS-Dep, DASS depression subscale; DASS-Anx, DASS anxiety subscale; DASS-Stress, DASS stress subscale.

### Analysis plan

To determine whether there were differences between OC users and non-users we first conducted a series of planned comparisons using *t*-tests comparing those using OCs and those not using OCs [a similar structure to the analyses of Raymond et al. (2019)] on each of our attention measures. While it was not one of the primary aims of the present study, we also compared depression, anxiety, and stress symptoms across OC and non-OC groups (for details on these analyses please see Supplementary Appendix C).

Further, given the relation between OC use and symptoms of depression (e.g., Keyes et al., 2013; Skovlund et al., 2016), we also sought to determine whether OC use could predict our attention measures over and above symptoms of depression. We also wanted to control for the semester of data collection since we were collecting data during the COVID-19 pandemic. To examine OC use while accounting for depression symptoms and the semester of data collection, we conducted a series of hierarchical regressions.

#### Planned comparisons

To examine whether there are differences in attention between OC users and non-users, we collapsed across semesters and conducted a series of independent sample *t*-tests (see Figure 1). These tests revealed that individuals using oral contraceptives reported significantly less spontaneous mind wandering (as measured by the MWS), *t*(809.4) = 3.18, *p* = 0.002, *d* = 0.17, less deliberate mind wandering (as measured by MWD), *t*(806.5) = 3.63, *p* < 0.001, *d* = 0.20, and fewer attention-related errors (which we measured using the ARCES), *t*(792.9) = 2.02, *p* = 0.044, *d* = 0.11^3^. There was no significant effect of contraceptive use on reports of attention lapses (as measured by the MAASLO), *t*(851.3) = 0.91, *p* = 0.362, *d* = 0.05.

Again, while it was not a primary goal of the present studies, we also investigated whether there were differences between OC users and non-users in their reports of symptoms of depression, anxiety, and stress. We again collapsed across semesters and conducted independent sample *t*-tests. Our findings indicated there was a significant difference in symptoms of depression, such that OC users reported significantly fewer depression symptoms compared to non-users. However, we found no significant differences between groups on anxiety or stress (for details please see Supplementary Appendix C).

#### Regressions

Given the relation between oral contraceptive use and symptoms of depression (e.g., Keyes et al., 2013; Skovlund et al., 2016), we also sought to determine whether oral contraceptive use could predict the attention measures over and above symptoms of depression and the semester of data collection, since data collection occurred during the COVID-19 pandemic. To this end, we conducted a series of hierarchical regressions entering semester and DASS-depression as predictors in the first step, and adding oral contraceptive use in the second step. For the sake of brevity, the R^2^ and ΔR^2^ associated with each regression are shown in Table 2; full regression tables are available in Supplementary Appendix B. As can be seen in Table 2, entering semester and depression symptoms in Step 1 accounted for a significant amount of variance in each measure of inattention. Critically, OC use explained significant additional variance over and above semester and depression in spontaneous and deliberate mind wandering, such that OC use uniquely predicted less spontaneous and deliberate mind wandering (MWS: b = -0.16, *p* = 0.019; MWD: b = -0.24, *p* = 0.003). However, we note that the amount of additional variance explained by OC use is quite small (e.g., less than 1%). OC use did not explain additional variance in attention related errors or attention lapses (see ΔR^2^ in Table 2).

**TABLE 2:** Regression model statistics for Study 1.

	R^2^	ΔR^2^	Model *p*	*P* for ΔR^2^
**DV: MWS**				
Step 1	0.146	–	<0.001	–
Step 2	0.149	0.003	<0.001	0.019
**DV: MWD**				
Step 1	0.039	–	<0.001	–
Step 2	0.044	0.005	<0.001	0.003
**DV: ARCES**				
Step 1	0.114	–	<0.001	–
Step 2	0.114	0.000	<0.001	0.268
**DV: MAASLO**				
Step 1	0.190	–	<0.001	–
Step 2	0.190	0.000	<0.001	0.761

DV, dependent variable; MWS, spontaneous mind wandering; MWD, deliberate mind wandering; MAASLO, mindful attention awareness scale; ARCES, attention-related cognitive errors scale. Semester of data collection and depression symptoms are entered in Step 1. In Step 2, oral contraceptive (OC) use is added to the model.

## Study 2

Contrary to prior work, our results from Study 1 indicated that there was no relation between OC use and reports of poorer attention in everyday life. In fact, our findings from this study indicated that OC use may provide a modest attentional benefit. Given this interesting result, we sought to determine whether this effect would replicate. To do this, we utilized the same method as Study 1 in an independent sample of undergraduate women.

### Methods

This study was also reviewed and received ethics clearance through a University of Waterloo Research Ethics Committee (ORE# 41701). The study was conducted in accordance with relevant guidelines and regulation. All participants provided informed consent. Similar to Study 1, data was collected online at the University of Waterloo at the beginning of three semesters during the 2021 calendar year: Winter 2021 (January-February), Spring 2021 (May-June), and Fall 2021 (September-October).

#### Prescreen

The prescreen procedure was identical to Study 1.

#### Data cleaning

The data cleaning procedure was identical to Study 1.

#### Materials

The materials were identical to those used in Study 1.

#### Participants

As in Study 1, all participants were female undergraduates, Testing in exchange for partial course credit, and exclusions were made using the criteria completed Mass outlined in the Data Cleaning section above. In Winter 2021, data was collected from 1,573 female participants and 1,042 were excluded (as a reminder, the number of exclusions is higher because a large number of participants were included in previous semesters). This resulted in 531 participants in Winter 2021, with 125 using OCs and 406 naturally cycling. Spring 2021 initially consisted of 490 participants but 355 were excluded leaving 135 participants with 23 OC users and 112 non-users. Fall 2021 began with data from 1,309 participants, of which 800 were excluded. After exclusions, Fall 2021 was composed of 509 participants: 98 using OCs and 411 naturally cycling. Thus, all together, Study 2 included a total of 1,175 participants with 246 OC users and 926 non-users.

Participants in Study 2 had an average age of 20.64 years old. Eighteen participants did not provide a birth year from which we could calculate their approximate age^4^. All participants in the study reported their sex as female. Participants also reported their gender identities. Most reported being cis-gendered females (*N* = 1155). Other gender identities included non-binary (*N* = 8), gender fluid (*N* = 1), and male (*N* = 2)^5^. Nine participants did not provide a gender identity.

## Results and discussion

Analyses were again performed in R Core Team (2019) using the same packages as Study 1. Anonymized data and analysis scripts will be made available at https://osf.io/dpxtn/.

### Descriptive statistics

**FIGURE 2:**
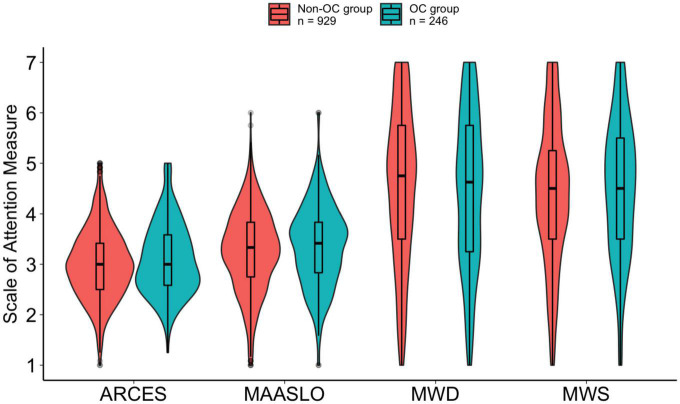
Violin plot with box and whisker plots (boxplots) for each of the attention measures [spontaneous mind wandering (MWS), deliberate mind wandering (MWD), mindful attention awareness scale (MAASLO), and attention-related cognitive errors scale (ARCES)] as a function of oral contraceptive (OC) group (Non-OC vs. OC) in Study 2.

Descriptive statistics for both groups are provided in Table 3. Scales again showed high reliabilities, with Cronbach alphas of 0.81 or greater (see Supplementary Appendix A3). We also include Pearson correlations between the measures within each group in Supplementary Appendix A4. Boxplots depicting each of the attention measures as a function of OC use condition (averaged across semesters) are shown in Figure 2.

**TABLE 3:** Descriptive statistics of measures by semester and group (Study 2; *N* = 1,175).

Semester	Group	Measure	*N*	Mean	SD	Skew	Kurtosis
Winter 2021	Non-OC group	ARCES	406	2.98	0.66	0.10	0.05
		MAASLO	406	3.23	0.82	–0.09	–0.07
		MWS	406	4.30	1.34	–0.15	–0.38
		MWD	406	4.42	1.56	–0.28	–0.72
		DASS-Dep	406	0.92	0.75	0.65	–0.47
		DASS-Anx	406	0.73	0.64	0.86	–0.04
		DASS-Stress	406	0.98	0.65	0.31	–0.75
	OC group	ARCES	125	3.06	0.67	0.52	0.25
		MAASLO	125	3.29	0.75	–0.60	0.32
		MWS	125	4.48	1.32	–0.09	–0.66
		MWD	125	4.48	1.52	–0.21	–0.71
		DASS-Dep	125	0.80	0.74	1.17	0.97
		DASS-Anx	125	0.70	0.69	1.02	0.17
		DASS-Stress	125	1.00	0.69	0.62	–0.32
Spring 2021	Non-OC group	ARCES	112	2.92	0.71	0.57	0.28
		MAASLO	112	3.33	0.80	0.10	0.29
		MWS	112	4.48	1.39	–0.04	–0.57
		MWD	112	4.46	1.47	–0.17	–0.42
		DASS-Dep	112	0.93	0.75	0.90	0.13
		DASS-Anx	112	0.71	0.66	1.13	0.86
		DASS-Stress	112	1.02	0.69	0.39	–0.51
	OC group	ARCES	23	3.13	0.66	0.00	–0.89
		MAASLO	23	3.55	0.85	–0.15	–1.17
		MWS	23	4.47	1.33	–0.78	0.95
		MWD	23	4.23	1.09	0.73	0.62
		DASS-Dep	23	0.83	0.66	1.37	2.03
		DASS-Anx	23	0.86	0.61	0.95	1.03
		DASS-Stress	23	1.12	0.53	1.17	1.35
Fall 2021	Non-OC group	ARCES	411	3.04	0.71	0.15	0.24
		MAASLO	411	3.27	0.78	–0.38	0.22
		MWS	411	4.45	1.45	–0.23	–0.36
		MWD	411	4.67	1.47	–0.38	–0.54
		DASS-Dep	411	0.89	0.72	0.74	–0.25
		DASS-Anx	411	0.82	0.66	0.87	0.21
		DASS-Stress	411	1.06	0.64	0.34	–0.63
	OC group	ARCES	98	3.13	0.77	0.48	–0.13
		MAASLO	98	3.41	0.85	0.50	0.50
		MWS	98	4.52	1.41	–0.33	–0.10
		MWD	98	4.50	1.62	–0.28	–0.91
		DASS-Dep	98	0.81	0.70	0.91	0.22
		DASS-Anx	98	0.83	0.65	0.63	–0.63
		DASS-Stress	98	1.09	0.63	0.41	–0.18

MWS, spontaneous mind wandering; MWD, deliberate mind wandering; MAASLO, mindful attention awareness scale; ARCES, attention-related cognitive errors scale; DASS-Dep, DASS depression subscale; DASS-Anx, DASS anxiety subscale, DASS-Stress, DASS stress subscale.

### Analysis plan

The Analysis Plan for Study 2 is identical to Study 1. We conducted planned comparisons of OC users to non-users and hierarchical regressions to determine whether OC use could predict our attention measures over and above the semester of data collection and symptoms of depression.

#### Planned comparisons

To investigate whether there were differences in our self-reported attention measures between those using OCs and those not using OCs, we again collapsed across semesters and conducted a series of independent samples *t*-tests (Figure 2). We did not find significant differences in spontaneous mind wandering (which we measured using the MWS), *t*(394.2) = 1.03, *p* = 0.304, *d* = 0.07, or deliberate mind wandering (as measured by the MWD), *t*(383.1) = 0.65, *p* = 0.519, *d* = 0.05. Our findings also indicated there were no significant differences in attention related errors (as measured by the ARCES), *t*(377.3) = 1.92, *p* = 0.055, *d* = 0.14 and no differences in attention lapses (indexed using the MAASLO), t(383.4) = 1.69, *p* = 0.092, *d* = 0.12.

While it was not a goal of the present study, we also examined whether symptoms of depression, anxiety, and stress differed between OC users and non-users by collapsing across semesters and conducting an independent samples *t*-tests. We found no significant differences between groups on symptoms of depression, anxiety, or stress. However, OC users reported numerically fewer symptoms of depression compared to non-users (*p* = 0.055), which is consistent with our findings from Study 1 (for details please see Supplementary Appendix C).

#### Regressions

As in Study 1, we sought to examine whether OC use uniquely predicted our attention measures over and above symptoms of depression. As before, we conducted a series of hierarchical regressions. In the first step of these regressions we entered semester and DASS-depression as predictors. In the second step we added contraceptive use (see Table 4; full regression tables are available in Supplementary Appendix B). Semester and depression symptoms together accounted for a significant amount of variance in each measure of inattention. However, the present findings differed from those in Study 1. OC use did not explain additional variance in spontaneous or deliberate mind wandering, however OC use did explain significant additional variance over and above semester and depression in attention related errors and attention lapses (ARCES: b = 0.14, *p* = 0.003; MAASLO: b = 0.15, *p* = 0.003), such that OC use uniquely predicted more attention related errors and more attention lapses (see ΔR^2^ in Table 4). However, once again, we note that the amount of additional variance explained by OC use is quite small (e.g., less than 1%).

**TABLE 4:** Regression model statistics for Study 2.

	R^2^	ΔR^2^	Model *p*	*P* for ΔR^2^
**DV: MWS**				
Step 1	0.127	–	<0.001	–
Step 2	0.130	0.003	<0.001	0.057
**DV: MWD**				
Step 1	0.041	–	<0.001	–
Step 2	0.041	0.000	<0.001	0.834
**DV: ARCES**				
Step 1	0.150	–	<0.001	–
Step 2	0.156	0.006	<0.001	0.003
**DV: MAASLO**				
Step 1	0.208	–	<0.001	–
Step 2	0.214	0.006	<0.001	0.003

DV, dependent variable; MWS, spontaneous mind wandering; MWD, deliberate mind wandering; MAASLO, mindful attention awareness scale; ARCES, attention-related cognitive errors scale. Semester of data collection and depression symptoms are entered in Step 1. In Step 2, oral contraceptive (OC) use is added to the model.

## General discussion

In two large samples, we did not find reliable and robust evidence to support the notion that OC use is detrimental to women’s attentional engagement in everyday life. In Study 1, our findings indicated a very modest opposite effect such that individuals using OCs reported slightly better attention (as indexed by reports of significantly *less* spontaneous and deliberate mind wandering and fewer attention-related errors) than those in the non-OC group. Regression analyses in Study 1 revealed that OC use predicted less spontaneous and deliberate mind wandering even when semester of data collection and depression were controlled; though this was not the case attention-related errors and attention lapses. While Study 2 was identical to Study 1, the results of Study 1 were not replicated in Study 2. Specifically, when controlling for semester of data collection and depression in Study 2, OC use did not explain additional variance in spontaneous and deliberate mind wandering. Moreover, after controlling for semester and depression in Study 2, OC users showed *more* attention-related errors and attention lapses than non-OC users. We draw particular attention to the small size of the effects revealed by the regression models, which appear to be unreliable and are unlikely to represent meaningful differences between groups in attentional engagement in everyday life. Our data also suggest there is considerable variability between cohorts and underscores the importance of collecting large samples when examining the relation between OC use and cognitive variables.

Our findings in Study 2 could be interpreted as being partially consistent with those reported by Raymond et al. (2019), who found that women using Ocs reported *more* mind wandering on one subscale of the SIPI than naturally cycling women. While we found that OC users showed *less* mind wandering than non-OC users (Study 1) or no difference from non-OC users (Study 2), our regression analyses in Study 2 showed that OC use was associated with more attention-related errors and lapses, which are constructs that are positively related to mind wandering. However, we hasten to highlight the inconsistency across studies even in the present analyses and the concerningly small effect sizes, which are likely related to the variability of outcomes across studies. Given the inconsistency between these two large samples, drawing strong conclusions about the relation between OC use and attention using smaller samples is likely problematic regardless of the measures used.

That being said, we should also note several ways in which our studies differed from those reported by Raymond et al. (2019). First, we used a different measure of mind wandering: We opted to measure mind wandering using the MWS and MWD, which measure participants’ general tendencies to spontaneously and deliberate mind wander without reference to specific situations. In contrast, Raymond et al. (2019) used the SIPI, which measures mind wandering in very specific contexts (e.g., while working).^6^ Given the different measurements employed, there is the possibility that each study captured different aspects of mind wandering. Second, with 28 women using OCs and 14 naturally cycling women, Raymond et al. (2019)’s study might not have been adequately powered to yield stable results. Because small sample sizes have been implicated as a “significant limitation” (Warren et al., 2014; p. 114) of many studies examining the relation between OC use and cognition, in the present study we aimed to recruit considerably larger samples than those used in previous work.

The present findings are worth considering in the context of the effects of OC use on various physiological systems. Particularly, prior research has linked OC use with elevated diurnal cortisol levels (Boisseau et al., 2013; Roche et al., 2013; Hill, 2019; p. 157) and a blunted free cortical response to acute stress (Kirschbaum et al., 1999; Boisseau et al., 2013; Roche et al., 2013; Hertel et al., 2017), both of which are indicators of chronic stress (Hertel et al., 2017). Chronic stress has a number of consequences—some of which may be detrimental to everyday attention and others which may be viewed as beneficial. For example, chronic stress has been linked with an increased risk of developing a mental health disorder (Tafet and Bernardini, 2003; Hammen, 2015), which can make attending to everyday tasks more challenging (Keller et al., 2019). Chronic stress can also lead to prolonged state of hyperarousal, which includes symptoms such as increased vigilance (e.g., de Quervain et al., 2017; Vargas et al., 2020), which could boost attention in everyday tasks. Thus, individual differences in the response to OC-induced chronic stress have the potential to lead to both reductions and improvements in everyday attention. This may explain at least some of the variability in results across studies.

There are several notable challenges when it comes to studying the influence of OC use that must be kept in mind. For example, one challenge inherent in most studies examining OC use is that in almost all such studies women are not randomly assigned to OC use and non-use groups (i.e., women self-select into groups). As such, it could be argued that differences in attention between OC users and non-users could be due to pre-existing differences in these cohorts that lead them to choose to be OC users or non-users. For instance, one might argue that since OCs require a prescription, women using OCs might have better access to health care and/or are more health conscious, and that by extension these individuals may have better cognitive health and attention. Researchers have examined this “health hypothesis” in cohort studies by comparing all-cause mortality rates of OC users and non-users. Most studies find no differences in all-cause mortality across OC and non-OC users (e.g., Beral et al., 1999; Vessey et al., 2010), with some work finding a protective effect of OC use against all-cause mortality compared to those who have never used OCs (Charlton et al., 2014). Thus, this work seems to suggest that OC users are just as healthy as non-OC users—though we note that all-cause mortality may not capture all aspects of healthy living. Importantly, any positive effects of OC use could also be partly explained in terms of a “survivor effect” whereby women who experience adverse effects of OCs might discontinue use (see Kay, 1984; Trussell and Kost, 1987; Chamberlain et al., 2020) and so be less likely to appear in the OC use condition in a study.

Finally, our work suggests several directions for future investigations. First, we collected self-reports of (or *perceptions* of) everyday attentional engagement. We did not collect behavioral measures of attentional engagement during a particular task. Self-reports are meta-cognitive judgments, and self-monitoring and memory can affect the accuracy of these reports (Koriat and Shitzer-Reichert, 2002). Future work could examine whether performance on an attentional task differs between OC users and non-users. Second, our analyses utilized secondary data and as such, we lacked details on participants current and historical OC use (e.g., the specific brand/generation of OCs used, length of use, whether non-users had used OCs in the past). Since different brands/generations of OCs have slightly different combinations of exogenous estrogen and progesterone (by dose and composition), some have noted that by collapsing across brand/generations it becomes impossible to learn whether there are effects specific to particular brands/generations (e.g., Wharton et al., 2008; Beltz et al., 2015). However, it is worth considering that splitting the OC group by brand/generation can result in small sample sizes in the resulting groups, which may lower statistical power. Indeed, while power analyses have demonstrated that a between subjects design requires 128 participants (64 participants per group) to detect a medium sized effect (Faul et al., 2007), few studies examining OC use and cognition reach this threshold to begin with—let alone after splitting the OC group with this specificity (see Warren et al., 2014; Gurvich et al., 2023). Nonetheless, future work could recruit participants using a homogenous OC brand or generation to investigate whether there is a relation between a particular OC brand/generation and everyday attentional engagement. Lastly, given that we collected data during the COVID-19 pandemic, it is possible that these circumstances and associated restrictions affected participants’ reports of attentional engagement. One possibility is that individuals were more inattentive or distractible during the pandemic (Hicks et al., 2021) and this suppressed differences in attention between OC users and non-users. Future work could attempt to replicate our findings after most pandemic-related restrictions have been lifted.

In summary, we found little evidence that OC users have poorer everyday attentional abilities than do non-OC users. Our findings indicate that any differences between OC users and non-users are small and unlikely to translate into meaningful differences in attentional engagement between groups. Future research should assess the influence of OC use on other cognitive factors with well-powered studies and with a consideration of the problems of self-selection and survivor effects.

## Data availability statement

The data presented in the study are deposited in the Open Science Framework (OSF) repository. This data can be found here: https://osf.io/dpxtn/.

## Ethics statement

The studies involving human participants were reviewed and approved by University of Waterloo Research Ethics Committee (ORE #41701). The patients/participants provided their written informed consent to participate in this study.

## Author contributions

AS: conceptualization, formal analysis, methodology, data curation, writing—original draft, and writing—review and editing. JM-D: formal analysis, data curation, and writing—review and editing. DS: supervision, conceptualization, methodology, and writing—review and editing. All authors contributed to the article and approved the submitted version.

## Footnotes

1 To preserve the anonymization of the data, age was calculated by subtracting participant’s year of birth from the year of data collection (in Study 1, data was collected in 2020).

2 Given that some participants reported a gender identity other than female, it is possible that some of these participants use an exogenous hormones other than hormonal contraceptives. As such, we conducted our analyses with these participants included and excluded. The exclusion of these participants did not change our findings in a meaningful way (i.e., the effect sizes did not substantially change) and as such, we opted to include these participants.

3 When we only include participants with a gender identity of female, this test becomes non-significant: *t*(798.40) = 1.85, *p* = 0.064, *d* = 0.10. However, the effect size remains stable.

4 To ensure data remained anonymous, we again calculated age by subtracting the year of birth provided from the year of data collection (in Study 2, data was collected in 2021).

5 As we did in Study 1, given the possibility that participants identifying with a gender identity other than female may use exogenous hormones, we ran our analyses with these participants both included and excluded. Similar to Study 1, the statistical results and effect sizes did not substantially differ, as such we opted to keep these participants in our analyzed sample.

6 The SIPI includes items such as “I find it difficult to concentrate with the TV on,” “my mind seldom wanders from my work,” and “I find that I easily lose interest in things that I have to do.” This measure excludes mind wandering outside of the specific contexts listed (e.g., it would fail to capture difficulty concentrating in a noisy coffee shop, the tendency to daydream outside of work, and the loss of interest in leisure activities).
