# Supplementary material for: Examining the relation between oral contraceptive use and attentional engagement in everyday life
Source: Front Hum Neurosci. 2023 Jun 1;17:1147515. doi: 10.3389/fnhum.2023.1147515 (PMC10267369; doi:10.3389/fnhum.2023.1147515)
Supplement: Supplementary file 1 [file Table_1.DOCX]

**Supplementary Materials**

**Appendix A**

**Study 1**

*Table A1 Cronbach alphas*

| *Measure* | *OC group*  *(N = 471)* | *Non-OC group*  *(N = 1330)* |
| --- | --- | --- |
| 1. MW-S | .89 | .88 |
| 2. MW-D | .92 | .90 |
| 3. MAAS-LO | .87 | .87 |
| 4. ARCES | .90 | .89 |
| 5.DASS-Depression | .90 | .90 |
| 6. DASS-Anxiety | .75 | .79 |
| 7. DASS-Stress | .84 | .84 |

*Note*: MW-S = Spontaneous Mind Wandering, MW-D = Deliberate Mind Wandering, MAAS-LO = Mindful Attention Awareness Scale; ARCES = Attention-Related Cognitive Errors Scale.

*Table A2 Pearson correlations of the OC group (above the diagonal; N=471) and non-OC group (below the diagonal; N = 1330)*

| *Measure* | *1* | *2* | *3* | *4* | *5* | *6* | *7* |
| --- | --- | --- | --- | --- | --- | --- | --- |
| 1. MW-S | -- | .51 ** | .49 ** | .55 ** | .34 ** | .32 ** | .35 ** |
| 2. MW-D | .40 ** | -- | .26 ** | .31 ** | .18 ** | .15 * | .11 * |
| 3. MAAS-LO | .52 ** | .26 ** | -- | .52 ** | .45 ** | .41 ** | .46 ** |
| 4. ARCES | .52 ** | .23 ** | .59 ** | -- | .31 ** | .39 ** | .41 ** |
| 5.DASS-Depression | .38 ** | .14 ** | .42 ** | .33 ** | -- | .59 ** | .70 ** |
| 6. DASS-Anxiety | .30 ** | .10 ** | .39 ** | .33 ** | .63 ** | -- | .72 ** |
| 7. DASS-Stress | .37 ** | .11 ** | .44 ** | .36 ** | .70 ** | .74 ** | -- |

*Note*: MW-S = Spontaneous Mind Wandering, MW-D = Deliberate Mind Wandering, MAAS-LO = Mindful Attention Awareness Scale; ARCES = Attention-Related Cognitive Errors Scale.

* p = .05, ** p < .001

**Study 2**

*Table A3 Cronbach alphas*

| *Measure* | *OC group*  *(N = 246)* | *Non-OC group*  *(N = 929)* |
| --- | --- | --- |
| 1. MW-S | .87 | .89 |
| 2. MW-D | .90 | .90 |
| 3. MAAS-LO | .88 | .87 |
| 4. ARCES | .90 | .90 |
| 5.DASS-Depression | .90 | .90 |
| 6. DASS-Anxiety | .82 | .81 |
| 7. DASS-Stress | .82 | .83 |

*Note*: MW-S = Spontaneous Mind Wandering, MW-D = Deliberate Mind Wandering, MAAS-LO = Mindful Attention Awareness Scale; ARCES = Attention-Related Cognitive Errors Scale.

*Table A4 Pearson correlations of the OC group (above the diagonal; N=246) and non-OC group (below the diagonal; N = 929)*

| *Measure* | *1* | *2* | *3* | *4* | *5* | *6* | *7* |
| --- | --- | --- | --- | --- | --- | --- | --- |
| 1. MW-S | -- | .44 ** | .46 ** | .50 ** | .28 ** | .31 ** | .37 ** |
| 2. MW-D | .44 ** | -- | .17 * | .21 * | .17 * | .18 * | .17 * |
| 3. MAAS-LO | .49 ** | .26 ** | -- | .60 ** | .42 ** | .49 ** | .52 ** |
| 4. ARCES | .51 ** | .20 ** | .55 ** | -- | .40 ** | .44 ** | .40 ** |
| 5.DASS-Depression | .37 ** | .19 ** | .47 ** | .38 ** | -- | .65 ** | .76 ** |
| 6. DASS-Anxiety | .34 ** | .14 ** | .42 ** | .38 ** | .60 ** | -- | .79 ** |
| 7. DASS-Stress | .38 ** | .18 ** | .49 ** | .38 ** | .69 ** | .73 ** | -- |

*Note*: MW-S = Spontaneous Mind Wandering, MW-D = Deliberate Mind Wandering, MAAS-LO = Mindful Attention Awareness Scale; ARCES = Attention-Related Cognitive Errors Scale.

* p = .05, ** p < .001

**Appendix B**

**Study 1**

**Regressions**

As noted in the manuscript, full regression model statistics for Study 1 are presented below.

Table B1 Regression predicting spontaneous mind wandering (*N* = 1801) by the term, DASS-Depression, and oral contraceptive use

| Predictors | R^2^ | ΔR^2^ | *F* | SE | b | *t* | *p* |
| --- | --- | --- | --- | --- | --- | --- | --- |
| *Step 1* | **.146** |  | **102.40** | **1.27** |  |  | **< .001** |
| Constant |  |  |  |  | 4.27 | 78.43 | < .001 |
| Term - Spring |  |  |  |  | -0.24 | 2.80 | .005 |
| Term - Fall |  |  |  |  | 0.02 | 0.25 | .806 |
| DASS-Depression |  |  |  |  | 0.51 | 17.01 | < .001 |
| *Step 2* | **.149** | **.003** | **78.37** | **1.27** |  |  | **< .001** |
| Constant |  |  |  |  | 4.32 | 74.17 | < .001 |
| Term - Spring |  |  |  |  | -0.25 | 2.85 | .004 |
| Term - Fall |  |  |  |  | 0.00 | 0.07 | .946 |
| DASS-Depression |  |  |  |  | 0.51 | 16.87 | < .001 |
| Birth control – OC use |  |  |  |  | -0.16 | 2.35 | .019 |

*Note.* The statistics for the overall model can be found in bold, to the right of Step 1 and Step 2 in the table above. Statistics for the model change can be found in-text in the results section of the manuscript.

Table B2 Regression predicting deliberate mind wandering (*N* = 1801) by the term, DASS-Depression, and oral contraceptive use

| Predictors | R^2^ | ΔR^2^ | *F* | SE | b | *t* | *p* |
| --- | --- | --- | --- | --- | --- | --- | --- |
| *Step 1* | **.039** |  | **24.26** | **1.49** |  |  | **< .001** |
| Constant |  |  |  |  | 4.26 | 66.50 | < .001 |
| Term - Spring |  |  |  |  | -0.18 | 1.78 | .076 |
| Term - Fall |  |  |  |  | 0.28 | 3.49 | < .001 |
| DASS-Depression |  |  |  |  | 0.22 | 6.31 | < .001 |
| *Step 2* | **.044** | **.005** | **20.55** | **1.49** |  |  | **< .001** |
| Constant |  |  |  |  | 4.33 | 63.31 | < .001 |
| Term - Spring |  |  |  |  | -0.19 | 1.84 | .066 |
| Term - Fall |  |  |  |  | 0.26 | 3.26 | .001 |
| DASS-Depression |  |  |  |  | 0.22 | 6.14 | < .001 |
| Birth control – OC use |  |  |  |  | -0.24 | 3.01 | .003 |

*Note.* The statistics for the overall model can be found in bold, to the right of Step 1 and Step 2 in the table above. Statistics for the model change can be found in-text in the results section of the manuscript.

Table B3 Regression predicting attention-related errors (*N* = 1801) by the term, DASS-Depression, and oral contraceptive use

| Predictors | R^2^ | ΔR^2^ | *F* | SE | b | *t* | *p* |
| --- | --- | --- | --- | --- | --- | --- | --- |
| *Step 1* | **.114** |  | **76.78** | **0.62** |  |  | **< .001** |
| Constant |  |  |  |  | 2.94 | 110.61 | < .001 |
| Term - Spring |  |  |  |  | -0.11 | 2.48 | .013 |
| Term - Fall |  |  |  |  | 0.06 | 1.68 | .094 |
| DASS-Depression |  |  |  |  | 0.21 | 14.32 | < .001 |
| *Step 2* | **.114** | **.000** | **57.90** | **0.62** |  |  | **< .001** |
| Constant |  |  |  |  | 2.95 | 103.68 | < .001 |
| Term - Spring |  |  |  |  | -0.11 | 2.51 | .012 |
| Term - Fall |  |  |  |  | 0.05 | 1.59 | .112 |
| DASS-Depression |  |  |  |  | 0.21 | 14.24 | < .001 |
| Birth control – OC use |  |  |  |  | -0.04 | 1.11 | .268 |

*Note.* The statistics for the overall model can be found in bold, to the right of Step 1 and Step 2 in the table above. Statistics for the model change can be found in-text in the results section of the manuscript.

Table B4 Regression predicting attention lapses (*N* = 1801) by the term, DASS-Depression, and oral contraceptive use

| Predictors | R^2^ | ΔR^2^ | *F* | SE | b | *t* | *p* |
| --- | --- | --- | --- | --- | --- | --- | --- |
| *Step 1* | **.190** |  | **140.60** | **0.70** |  |  | **< .001** |
| Constant |  |  |  |  | 3.23 | 107.91 | < .001 |
| Term - Spring |  |  |  |  | -0.17 | 3.48 | < .001 |
| Term - Fall |  |  |  |  | 0.00 | 0.01 | .989 |
| DASS-Depression |  |  |  |  | 0.33 | 19.93 | < .001 |
| *Step 2* | **.190** | **.000** | **105.4** | **0.70** |  |  | **< .001** |
| Constant |  |  |  |  | 3.23 | 100.62 | < .001 |
| Term - Spring |  |  |  |  | -0.17 | 3.47 | < .001 |
| Term - Fall |  |  |  |  | 0.00 | 0.04 | .970 |
| DASS-Depression |  |  |  |  | 0.33 | 19.91 | < .001 |
| Birth control – OC use |  |  |  |  | 0.01 | 0.30 | .761 |

*Note.* The statistics for the overall model can be found in bold, to the right of Step 1 and Step 2 in the table above. Statistics for the model change can be found in-text in the results section of the manuscript.

**Study 2**

**Regressions**

Like Study 1 above, we present full regression model statistics for Study 2 below.

Table B5 Regression predicting spontaneous mind wandering (*N* = 1175) by the term, DASS-Depression, and oral contraceptive use

| Predictors | R^2^ | ΔR^2^ | *F* | SE | b | *t* | *p* |
| --- | --- | --- | --- | --- | --- | --- | --- |
| *Step 1* | **.127** |  | **56.81** | **1.30** |  |  | **< .001** |
| Constant |  |  |  |  | 4.34 | 77.03 | < .001 |
| Term - Spring |  |  |  |  | 0.12 | 0.98 | .330 |
| Term - Fall |  |  |  |  | 0.13 | 1.64 | .102 |
| DASS-Depression |  |  |  |  | 0.49 | 12.96 | < .001 |
| *Step 2* | **.130** | **.003** | **43.61** | **1.30** |  |  | **< .001** |
| Constant |  |  |  |  | 4.30 | 71.14 | < .001 |
| Term - Spring |  |  |  |  | 0.13 | 1.07 | .287 |
| Term - Fall |  |  |  |  | 0.14 | 1.73 | .084 |
| DASS-Depression |  |  |  |  | 0.49 | 13.05 | < .001 |
| Birth control – OC use |  |  |  |  | 0.17 | 1.90 | .057 |

*Note.* The statistics for the overall model can be found in bold, to the right of Step 1 and Step 2 in the table above. Statistics for the model change can be found in-text in the results section of the manuscript.

Table B6 Regression predicting deliberate mind wandering (*N* = 1175) by the term, DASS-Depression, and oral contraceptive use

| Predictors | R^2^ | ΔR^2^ | *F* | SE | b | *t* | *p* |
| --- | --- | --- | --- | --- | --- | --- | --- |
| *Step 1* | **.041** |  | **16.75** | **1.49** |  |  | **< .001** |
| Constant |  |  |  |  | 4.43 | 68.62 | < .001 |
| Term - Spring |  |  |  |  | -0.02 | 0.14 | .893 |
| Term - Fall |  |  |  |  | 0.21 | 2.32 | .020 |
| DASS-Depression |  |  |  |  | 0.29 | 6.68 | < .001 |
| *Step 2* | **.041** | **.000** | **12.57** | **1.49** |  |  | **< .001** |
| Constant |  |  |  |  | 4.43 | 63.97 | < .001 |
| Term - Spring |  |  |  |  | -0.02 | 0.14 | .885 |
| Term - Fall |  |  |  |  | 0.21 | 2.31 | .021 |
| DASS-Depression |  |  |  |  | 0.29 | 6.65 | < .001 |
| Birth control – OC use |  |  |  |  | -0.02 | 0.21 | .834 |

*Note.* The statistics for the overall model can be found in bold, to the right of Step 1 and Step 2 in the table above. Statistics for the model change can be found in-text in the results section of the manuscript.

Table B7 Regression predicting attention related errors (*N* = 1175) by the term, DASS-Depression, and oral contraceptive use

| Predictors | R^2^ | ΔR^2^ | *F* | SE | b | *t* | *p* |
| --- | --- | --- | --- | --- | --- | --- | --- |
| *Step 1* | **.150** |  | **68.91** | **0.64** |  |  | **< .001** |
| Constant |  |  |  |  | 3.00 | 107.52 | < .001 |
| Term - Spring |  |  |  |  | -0.05 | 0.77 | .441 |
| Term - Fall |  |  |  |  | 0.06 | 1.60 | .111 |
| DASS-Depression |  |  |  |  | 0.27 | 14.26 | < .001 |
| *Step 2* | **.156** | **.006** | **54.21** | **0.64** |  |  | **< .001** |
| Constant |  |  |  |  | 2.97 | 99.41 | < .001 |
| Term - Spring |  |  |  |  | -0.04 | 0.63 | .528 |
| Term - Fall |  |  |  |  | 0.07 | 1.75 | .081 |
| DASS-Depression |  |  |  |  | 0.27 | 14.45 | < .001 |
| Birth control – OC use |  |  |  |  | 0.14 | 2.95 | .003 |

*Note.* The statistics for the overall model can be found in bold, to the right of Step 1 and Step 2 in the table above. Statistics for the model change can be found in-text in the results section of the manuscript.

Table B8 Regression predicting attention lapses (*N* = 1175) by the term, DASS-Depression, and oral contraceptive use

| Predictors | R^2^ | ΔR^2^ | *F* | SE | b | *t* | *p* |
| --- | --- | --- | --- | --- | --- | --- | --- |
| *Step 1* | **.208** |  | **102.6** | **0.71** |  |  | **< .001** |
| Constant |  |  |  |  | 3.24 | 104.81 | < .001 |
| Term - Spring |  |  |  |  | 0.11 | 1.67 | .095 |
| Term - Fall |  |  |  |  | 0.06 | 1.41 | .159 |
| DASS-Depression |  |  |  |  | 0.36 | 17.44 | < .001 |
| *Step 2* | **.214** | **.006** | **79.73** | **0.71** |  |  | **< .001** |
| Constant |  |  |  |  | 3.21 | 96.87 | < .001 |
| Term - Spring |  |  |  |  | 0.13 | 1.82 | .069 |
| Term - Fall |  |  |  |  | 0.07 | 1.56 | .118 |
| DASS-Depression |  |  |  |  | 0.37 | 17.64 | < .001 |
| Birth control – OC use |  |  |  |  | 0.15 | 3.00 | .003 |

*Note.* The statistics for the overall model can be found in bold, to the right of Step 1 and Step 2 in the table above. Statistics for the model change can be found in-text in the results section of the manuscript.

**Appendix C**

**Study 1**

**DASS Scores**

To determine if there were differences between groups on symptoms of depression, anxiety, and stress, we conducted a series of independent samples t-tests. We found there was a significant differences between groups on symptoms of depression, *t*(868.8) = 2.65, *p* = .008, *d* = .14, such that those using OCs reported significantly fewer depression symptoms than non-OC users. There were no differences between groups on either symptoms of anxiety, *t*(843.6) = 1.19, *p* = .234, *d* = .06, or symptoms of stress, *t*(818.1) = 0.51, *p* = .612, *d* = .03.

**Study 2**

**DASS Scores**

To investigate whether there were differences in symptoms of depression, anxiety, and stress between those using OCs and those not, we conducted a series of independent samples t-tests. We found no significant differences between groups on depression symptoms, *t*(393.6) = 1.93, *p* = .055, *d* = .14 (we note that OC users reported nominally fewer depression symptoms than non-users). We also did not find differences in symptoms of anxiety, *t*(379.2) = 0.04, *p* = .972, *d* = .00, nor symptoms of stress, *t*(382.5) = 0.61, *p* = .540, *d* = .04.
